# Supplementary material for: Genetic characterization of Bacillus anthracis strains circulating in Italy from 1972 to 2018
Source: PLoS One. 2020 Jan 13;15(1):e0227875. doi: 10.1371/journal.pone.0227875 (PMC6957342; doi:10.1371/journal.pone.0227875)

PLOS ONE

Genetic characterization of Bacillus anthracis strains circulating in Italy from 1972 to 2018

--Manuscript Draft--

**Manuscript Number:**	PONE-D-19-23489
**Article Type:**	Research Article
**Full Title:**	Genetic characterization of Bacillus anthracis strains circulating in Italy from 1972 to 2018
**Short Title:**	Genetic characterization of Bacillus anthracis in Italy
**Corresponding Author:**	Domenico Galante<br>Istituto Zooprofilattico Sperimentale della Puglia e della Basilicata<br>Foggia, ITALY
**Keywords:**	Bacillus anthracis; anthrax; molecular epidemiology; CanSNPs; MLVA; Italy
**Abstract:**	In Italy, anthrax is considered an endemic disease and almost every year a few outbreaks occur. In this survey, 234 B. anthracis strains from animals (n=196), humans (n=3) and the environment (n=35) isolated during Italian outbreaks from 1972 to 2018 have been analyzed. Despite the great genetic homogeneity of this bacterium, the canonical single nucleotide polymorphisms assay (CanSNPs) and the multiple-locus variable-number tandem repeat analysis (MLVA) have resulted to be highly effective to differentiate strains. The phylogenetic identity was determined through the research of polymorphisms for CanSNPs, with a set of 14 SNPs, while a 31-loci MLVA assay was performed to determine B. anthracis genotypes. The analysis of 14 CanSNPs, allowed to identify four main lineages: A.Br.011/009, A.Br. 008/011 (respectively belonging to A.Br. 008/009 sub-lineage, also known Trans-Eurasian or TEA group), A.Br. 005/006 and B. Br. CNEVA. The lineage A major subgroup A.Br.011/009 represents the major genotype of B. anthracis in Italy. The MLVA with 31 VNTRs analysis, demonstrated 55 different genotypes circulating in Italy. Most of them were genetically very similar to each other, confirming the hypothesis that all of them are the result of the evolution of a local common ancestral strain, except for two genotypes belonging to the branch A.Br. 005/006 and B. Br. CNEVA respectively. The genotyping analysis with methods such as CanSNPs and MLVA, remains a very valuable tool for studying the diversity, evolution, and molecular epidemiology of B. anthracis.
**Order of Authors:**	Domenico Galante
	Luigina Serrecchia
	Antonio Parisi
	Antonio Fasanella
	Viviana Manzulli
	Dora Cipolletta
	Valeria Rondinone
**Additional Information:**	
**Question**	**Response**
**Financial Disclosure**<br><br>Enter a financial disclosure statement that describes the sources of funding for the work included in this submission. Review the submission guidelines for detailed requirements. View published research articles from *PLOS ONE* for specific	A.F.<br>The study was funded by Italian Ministry of Health, by a research program "Ricerca corrente IZS PB 005/15 RC".<br>www.salute.gov.it<br>The funders had no role in study design, data collection and analysis, decision to publish, or preparation of the manuscript.

Powered by Editorial Manager® and ProduXion Manager® from Aries Systems Corporation

examples.<br><br>This statement is required for submission and **will appear in the published article** if the submission is accepted. Please make sure it is accurate.<br><br>**Unfunded studies**<br>Enter: *The author(s) received no specific funding for this work.*<br><br>**Funded studies**<br>Enter a statement with the following details:<br>• Initials of the authors who received each award<br>• Grant numbers awarded to each author<br>• The full name of each funder<br>• URL of each funder website<br>• Did the sponsors or funders play any role in the study design, data collection and analysis, decision to publish, or preparation of the manuscript?<br>• **NO** - Include this sentence at the end of your statement: *The funders had no role in study design, data collection and analysis, decision to publish, or preparation of the manuscript.*<br>• **YES** - Specify the role(s) played.<br><br>* typeset	
**Competing Interests**<br><br>Use the instructions below to enter a competing interest statement for this submission. On behalf of all authors, disclose any competing interests that could be perceived to bias this work—acknowledging all financial support and any other relevant financial or non-financial competing interests.<br><br>This statement **will appear in the published article** if the submission is accepted. Please make sure it is accurate. View published research articles from *PLOS ONE* for specific examples.	The authors have declared that no competing interests exist.

Powered by Editorial Manager® and ProduXion Manager® from Aries Systems Corporation

**NO authors have competing interests**<br><br>Enter: *The authors have declared that no competing interests exist.*<br><br>**Authors with competing interests**<br><br>Enter competing interest details beginning with this statement:<br><br>*I have read the journal's policy and the authors of this manuscript have the following competing interests: [insert competing interests here]*<br><br>* typeset	
**Ethics Statement**<br><br>Enter an ethics statement for this submission. This statement is required if the study involved:<br><br>• Human participants<br>• Human specimens or tissue<br>• Vertebrate animals or cephalopods<br>• Vertebrate embryos or tissues<br>• Field research<br><br>Write "N/A" if the submission does not require an ethics statement.<br><br>General guidance is provided below. Consult the submission guidelines for detailed instructions. **Make sure that all information entered here is included in the Methods section of the manuscript.**	N/A

**Format for specific study types**<br><br>**Human Subject Research (involving human participants and/or tissue)**<br>• Give the name of the institutional review board or ethics committee that approved the study<br>• Include the approval number and/or a statement indicating approval of this research<br>• Indicate the form of consent obtained (written/oral) or the reason that consent was not obtained (e.g. the data were analyzed anonymously)<br><br>**Animal Research (involving vertebrate animals, embryos or tissues)**<br>• Provide the name of the Institutional Animal Care and Use Committee (IACUC) or other relevant ethics board that reviewed the study protocol, and indicate whether they approved this research or granted a formal waiver of ethical approval<br>• Include an approval number if one was obtained<br>• If the study involved *non-human primates*, add *additional details* about animal welfare and steps taken to ameliorate suffering<br>• If anesthesia, euthanasia, or any kind of animal sacrifice is part of the study, include briefly which substances and/or methods were applied<br><br>**Field Research**<br><br>Include the following details if this study involves the collection of plant, animal, or other materials from a natural setting:<br>• Field permit number<br>• Name of the institution or relevant body that granted permission	
**Data Availability**<br><br>Authors are required to make all data underlying the findings described fully available, without restriction, and from the time of publication. PLOS allows rare exceptions to address legal and ethical concerns. See the PLOS Data Policy and FAQ for detailed information.	Yes - all data are fully available without restriction

Powered by Editorial Manager® and ProduXion Manager® from Aries Systems Corporation

A Data Availability Statement describing where the data can be found is required at submission. Your answers to this question constitute the Data Availability Statement and **will be published in the article**, if accepted.<br><br>**Important:** Stating 'data available on request from the author' is not sufficient. If your data are only available upon request, select 'No' for the first question and explain your exceptional situation in the text box.<br><br>Do the authors confirm that all data underlying the findings described in their manuscript are fully available without restriction?	
**Describe where the data may be found in full sentences. If you are copying our sample text, replace any instances of XXX with the appropriate details.**<br><br>• If the data are **held or will be held in a public repository**, include URLs, accession numbers or DOIs. If this information will only be available after acceptance, indicate this by ticking the box below. For example: *All XXX files are available from the XXX database (accession number(s) XXX, XXX.).*<br>• If the data are all contained **within the manuscript and/or Supporting Information files**, enter the following: *All relevant data are within the manuscript and its Supporting Information files.*<br>• If neither of these applies but you are able to provide **details of access elsewhere**, with or without limitations, please do so. For example:<br><br>*Data cannot be shared publicly because of [XXX]. Data are available from the XXX Institutional Data Access / Ethics Committee (contact via XXX) for researchers who meet the criteria for access to confidential data.*<br><br>*The data underlying the results presented in the study are available from (include the name of the third party*	All relevant data are within the manuscript and its Supporting Information files.

and contact information or URL).<br>• This text is appropriate if the data are owned by a third party and authors do not have permission to share the data.<br><br>* typeset	
Additional data availability information:	Tick here if the URLs/accession numbers/DOIs will be available only after acceptance of the manuscript for publication so that we can ensure their inclusion before publication.

Powered by Editorial Manager® and ProduXion Manager® from Aries Systems Corporation

Manuscript Click here to download Manuscript Manuscript.docx

Genetic characterization of *Bacillus anthracis* strains circulating in Italy from 1972 to 2018.

Valeria Rondinone [1], Luigina Serrecchia[1], Antonio Parisi [1], Antonio Fasanella [1], Viviana Manzulli [1], Dora Cipolletta [1], Domenico Galante [1]*

[1] *Experimental Zooprophylactic Institute of Apulia and Basilicata Regions, Anthrax Reference Institute of Italy, Foggia, Italy*

Short title: Genetic characterization of *Bacillus anthracis* in Italy

**Corresponding author:* Dr. Domenico Galante

Anthrax Reference Institute of Italy,

Experimental Zooprophylactic Institute of Puglia and Basilicata Regions

Via Manfredonia 20,71121, Foggia (FG), Italy

Tel.: +39 0881786330

E-mail: domenico.galante@izspb.it

Abstract

In Italy, anthrax is considered an endemic disease and almost every year a few outbreaks occur. In this survey, 234 *B. anthracis* strains from animals (n=196), humans (n=3) and the environment (n=35) isolated during Italian outbreaks from 1972 to 2018 have been analyzed. Despite the great genetic homogeneity of this bacterium, the canonical single nucleotide polymorphisms assay (CanSNPs) and the multiple-locus variable-number tandem repeat analysis (MLVA) have resulted to be highly effective to differentiate strains. The phylogenetic identity was determined through the research of polymorphisms for CanSNPs, with a set of 14 SNPs, while a 31-loci MLVA assay was performed to determine *B. anthracis* genotypes. The analysis of 14 CanSNPs, allowed to identify four main lineages: A.Br.011/009, A.Br. 008/011 (respectively belonging to A.Br. 008/009 sub-lineage, also known Trans-Eurasian or TEA group), A.Br. 005/006 and B. Br. CNEVA. The lineage A major subgroup A.Br.011/009 represents the major genotype of *B. anthracis* in Italy. The MLVA with 31 VNTRs analysis, demonstrated 55 different genotypes circulating in Italy. Most of them were genetically very similar to each other, confirming the hypothesis that all of them are the result of the evolution of a local common ancestral strain, except for two genotypes belonging to the branch A.Br. 005/006 and B. Br. CNEVA respectively. The genotyping analysis with methods such as CanSNPs and MLVA, remains a very valuable tool for studying the diversity, evolution, and molecular epidemiology of *B. anthracis*.

Introduction

Anthrax is a non-contagious infectious disease that affects a wide range of animal species including humans. *Bacillus anthracis*, the etiological agent of anthrax, forms long-lasting, highly resistant spores able to persist into environment for several decades [[illegible]]. Domestic and wild ruminants represent the most susceptible species [2].

Animals are infected during grazing in area contaminated with anthrax spores, while humans can contract the disease by contact with anthrax-infected animals or anthrax-contaminated animal products [3]. Three types of human anthrax occur, depending ~~of~~ exposure: cutaneous, which is usually non-fatal, gastrointestinal and inhalational which ~~are~~ both fatal [4]. Recently, a further fatal form was reported in drug users who have injected drugs contaminated with anthrax spores [5]. Since it is relatively easy and inexpensive to obtain, *B. anthracis* is one of the preferred pathogenic agents for use as bacteriological weapon in bio-terrorist attacks [6].

Usually in Italy, anthrax is a sporadic disease occurring particularly during the summer with a few exceptions, in the central and southern regions and in the major islands, where it almost exclusively affects animals at pasture [7].The risk of humans contracting anthrax is related to many factors, first of all the origin of the infection in the animal outbreak. From 1972 until 2018, about 200 outbreaks of animal anthrax were recorded (unpublished data). Rarely, however, anthrax occurs as epidemic-like disease, characterized by outbreaks involving a very limited area and a large number of animals. In Italy, where anthrax is hypo-enzootic, two major epidemic-like anthrax outbreaks occurred: during the summer of 2004 in Basilicata and during the summer 2011, in an area between Basilicata and Campania [8, 9].

Molecular tools, such as the canonical SNPs assay (CanSNPs) and multiple-locus variable-number tandem repeat analysis (MLVA), have proved to be highly effective to differentiate strains. In this study SNP analysis has been used to illustrate the phylogenetic relationship between *B. anthracis* strains and the 31-loci MLVA assay was performed to genotype *B. anthracis* isolates in order to

examine relationships among the Italian anthrax outbreaks and to assess the diversity of *B. anthracis* isolates related to regional and global scale.

Materials and Methods

Ethics statement

The animal and environmental strains used in this study were isolated by the Anthrax Reference Institute of Italy (Ce.R.N.A.). The Ce.R.N.A. is a public laboratory, mandated by the Italian Ministry of Health to confirm diagnosis of all animal anthrax cases in Italy. During outbreaks, samples are taken by the veterinary services of the Ministry of Health. Specific permission for soil sampling was not required. Human DNAs were also included in this study, and were received by the National Institute for Infectious Disease "L. Spallanzani" [10].

Bacterial Strains

A collection of 234 *B. anthracis* strains, including 196 isolated from animal species, 3 from humans and 35 from environment, isolated during Italian outbreaks from 1972 to 2018, were analyzed in this study as shown in Table 1.

Table 1. Summary of *B. anthracis* isolates from 1972 to 2018 analyzed in this study.

Sample type	Source	No. of isolates
Environmental samples	Water	3
	Soil	32
Animal samples	Bovine	101
	Caprine	20
	Deer	7
	Equine	12
	Ovine	53

	Swine	3
Human samples	Human	3

DNA Extraction

Each *B. anthracis* strains was seeded on 5% sheep blood agar plates and then incubated at +37°C for 24 hours. Microbial DNA was extracted using the DNAeasy Blood and Tissue kit (Qiagen, USA) following the protocol for Gram-positive bacteria. All the manipulations of *B. anthracis* strains were performed in a biosafety level 3 laboratory using class II type A 2 biosafety cabinet.

Real Time PCR assay

B. anthracis molecular identification was carried out by PCR using anthrax specific primers for the sequences located on plasmids pXO1 and pXO2 and primers specific for the chromosome [11].

CanSNP analysis

The canSNP profiles were obtained, using 13 allelic discrimination assays with oligonucleotides and probes as described by Van Ert et al.[1[illegible]].
Each 10 µl reaction contained 1× TaqMan Genotyping Master Mix (Applied Biosystems), 250 nM of each probe and 600 nM each of forward and reverse primers and approximately 10 ng of template DNA. For all assays, thermal cycling parameters were 95°C for 10 min, followed by 40 cycles of 95°C for 15 s and 60°C for 1 min. Endpoint fluorescent data were measured on the AB1 7900HT. The results obtained by CanSNPs were compared to the recognized 12 sub-lineage or sub-groups.
The 14th SNP was detected by High Resolution Melting (HRM) assays for specific A.Br. 011 CanSNP [13,14], using Precision Melt Analysis Software. The position based on the Ames Ancestor genome (NC_007530.2) is 2552486. Amplification was performed on CFX Connect Real-Time System (BIORAD) using the Precision Melt Supermix BIORAD. The reaction mixture consisted of

0.2 µM of each primer, 1x Precision Melt Supermix BIORAD in a 20 µl final volume. The following parameters were used: 2 min at 95°C were followed by 35 cycles consisting of 10 s at 95°C and 30 s at 60°C. Samples were next heated to 95°C for 30 s, cooled down to 60°C for 1 min and heated from 65°C to 95°C at rate of 0.5°C/s. HRM data were analyzed by Precision Melt Analysis Software.

31-loci MLVA analysis

To perform a 31-marker MLVA, we used 5' fluorescent-labeled oligos (6-FAM, VIC, NED and PET) ~~deprotected and desalted,~~ specifically selected for the Variable Number Tandem Repeats (VNTRs). Twenty-seven VNTRs loci present at the level of the chromosome (vrrA, vrrB1, cg3, vrrB2, vntr19, vrrC1, vrrC2,vntr32, vntr12, vntr35, vntr23bams03, bams05, bams13, bams15, bams21, bams22, bams23, bams24, bams25, bams28, bams30, bams31, bams34, bams44, bams51, bams53) and four at the level of plasmids (vntr16, vntr17, pxO1, pxO2)[12, 15, 16, 17, 18]. MLVA test consisted in the preparation of two Singleplex and nine Multiplex reactions, in a final volume of 15 µl. Each reaction mixture contained: 1X PCR reaction buffer (Qiagen); 3 mM $MgCl_2$, 0.2mM for each dNTPs; 1UI Hot Star Plus Taq DNA polymerase (Qiagen) and appropriate concentrations of each primer (singleplex 1: vrrC1, 0.2 µM; singleplex 2: vrrC2,0.2µM;multiplex 1:vrrA, 0.2 µM; vrrB1, 0.2µM and CG3, 0.4 µM; multiplex 2: vrrB2, 0.25 µM; pXO2, 0.1 µM; pXO1, 0.3 µM; multiplex 3: vntr12, 0.25 µM; vntr19,0.2 µM; vntr35, 0.2 µM; multiplex 4: vntr16, 0.25 µM; vntr23, 0.2 µM; multiplex 5: vntr17, 0.1 µM; vntr32, 0.4 µM; multiplex 6: bams03,0.8 µM; bams05, 0.2µM; bams15, 0.5µM and bams44, 0.05 µM; multiplex 7: bams21, 0.4 µM, bams24 and bams25, 0.3 µM, bams34, 0.2µM; multiplex 8: bams13, 0.3 µM, bams28, 0.15µM, bams31and bams53, 0.6 µM; multiplex 9: bams23, 0.2 µM, bams30, 0.6 µM, bams22 and bams51, 0.3 µM; and 2 µl of DNA.

The PCR thermocycling program for two singleplex and Multiplex 1 and 2 was the same: 95°C for 5 minutes; 35 cycles at 94°C for 30 seconds, at 60°C for 30 seconds, and 72°C for 30 seconds. The final step was at 72°C for 5 min.

The amplification program for another multiplex was the following: 95°C for 5 minutes, 35 cycles to 94°C for 30 seconds, 54°C for 30 seconds, 72°C for 45 seconds and 72°C for 5 minutes (multiplex 3); 95°C for 5 minutes, 35 cycles at 94°C for 30 seconds, 56°C for 45 seconds, 72°C for 1 minute, and 72° for 5 minutes (multiplex 4); 95°C for 5 minutes, 35 cycles at 94°C for 30 seconds, 59°C for 45 seconds, 72°C for 1 minute and 72°C for 5 minutes (multiplex 5).

The Multiplex-PCRs from 6 to 9 were carried out with the following profile:

Denaturation at 94°C, 5 min; 35 cycles with denaturation at 94°C, 1 min; annealing at 60°C, 90 sec; elongation at 72°C, 90 sec; final elongation step at 72°C, 15 min; and cooling at 4°C.

Automated genotype analysis

The MLVA PCR products were diluted 1:80 and subjected to capillary electrophoresis on ABI Prism 3130 genetic analyzer (Applied Biosystems) with 0.25 µl of GeneScan 1200 and sized by Gene Mapper 4.0 (Applied Biosystems Inc.).

The assignment of the sizes and the corresponding repeating unit numbers for each locus was carried out using as reference the strain Ames Ancestor accession NC_007530.2 (Chromosome), Nc_007322.2 (plasmid pXO1) and NC_007323.2 (plasmid pXO2) and attributing the conventional values proposed in the updated version of the 2016 *Bacillus anthracis* MLVAdatabase and available at MLVAbank http://mlva.u-psud.fr/.

Results

Real Time PCR assay

All the analyzed strains resulted PCR positive for chromosomal and plasmids pXO1 (codifying for toxins) and pXO2 (capsule formation) targets.

Canonical Single Nucleotide Polimorphisms (CanSNPs)

The analysis of the 13 classical CanSNPs, demonstrated that 231 strains belong to sublineage A.Br. 008/009 or TEA group, that it is established in southern and eastern Europe and represents the dominant subgroup in Italy, Bulgaria, Hungary and Albania [7, 12, 19, 20, 21]. However, the analysis of the additional 14th CanSNP (A.Br.011), recently allowed to divide the A.Br. 008/009 group into 2 subgroups and showed that 207 strains belonged to the main sub-lineage A.Br. 011/009 and 24 to the sub-lineage A.Br. 008/011. Twenty-three out of 24 strains belonging to the sub-lineage A.Br. 008/011 were isolated in Sicily and 1 in Umbria.

One strain isolated in Veneto belonged to the main lineage A, sub-lineage A.Br. 005/006, while other 2 strains, 1 isolated in Veneto and 1 isolated in Trentino, belonged to the main lineage B, sub-lineage B.Br. CNEVA.

Multiple Locus Variable Number Tandem Repeat with 31 VNTRs (MLVA)

The MLVA with 31 VNTRs analysis, identified 55 different genotypes distributed in the Italian regions named from GT-1 to GT-55 (Fig 1). The GT-14 resulted to be the most represented one and included 34 strains distributed mostly in Basilicata, Apulia and Calabria. To the GT-31 belonged 19 isolates: 16 from Tuscany, 2 from Apulia and 1 from Sardinia. The GT-26 and the GT-27 were

isolated just from Basilicata and Campania Regions. Other unique genotypes were characteristic for single Regions as showed in Table 2.

Fig 1. Geographical distribution of the 55 *B. anthracis* genotypes circulating in Italy

Table 2. Distribution of *B. anthracis* CanSNPs and genotypes circulating in Italy from 1972 to 2018.

Number of isolates	Regions	CanSNPs sublineage	Genotype
**1**	Apulia	A.Br. 011/009	MLVA31-1
**1**	Apulia	A.Br. 011/009	MLVA31-2
**1**	Apulia	A.Br. 011/009	MLVA31-3
**3**	Campania	A.Br. 011/009	MLVA31-4
**1**	Sardinia	A.Br. 011/009	MLVA31-5
**3**	Sardinia	A.Br. 011/009	MLVA31-6
**2**	Apulia	A.Br. 011/009	MLVA31-7
**1**	Umbria	A.Br. 008/011	MLVA31-8
**14**	Tuscany	A.Br. 011/009	MLVA31-9
**3**	Sicily	A.Br. 011/009	MLVA31-10
**1**	Tuscany	A.Br. 011/009	MLVA31-11
**3**	Sicily	A.Br. 011/009	MLVA31-12
**1**	Lombardy	A.Br. 011/009	MLVA31-13
**34**	Basilicata/Apulia/Calabria	A.Br. 011/009	MLVA31-14
**1**	Apulia	A.Br. 011/009	MLVA31-15
**2**	Apulia	A.Br. 011/009	MLVA31-16
**1**	Apulia	A.Br. 011/009	MLVA31-17
**1**	Basilicata	A.Br. 011/009	MLVA31-18
**1**	Apulia	A.Br. 011/009	MLVA31-19
**1**	Apulia	A.Br. 011/009	MLVA31-20
**1**	Apulia	A.Br. 011/009	MLVA31-21
**1**	Apulia	A.Br. 011/009	MLVA31-22
**1**	Apulia	A.Br. 011/009	MLVA31-23
**57**	Basilicata	A.Br. 011/009	MLVA31-24

**3**	Basilicata	A.Br. 011/009	MLVA31-25
**3**	Campania/Basilicata	A.Br. 011/009	MLVA31-26
**9**	Campania/Basilicata	A.Br. 011/009	MLVA31-27
**5**	Basilicata	A.Br. 011/009	MLVA31-28
**1**	Apulia	A.Br. 011/009	MLVA31-29
**1**	Sardinia	A.Br. 011/009	MLVA31-30
**19**	Tuscany/Apulia/Sardinia	A.Br. 011/009	MLVA31-31
**1**	Apulia	A.Br. 011/009	MLVA31-32
**1**	Apulia	A.Br. 011/009	MLVA31-33
**5**	Apulia	A.Br. 011/009	MLVA31-34
**6**	Apulia	A.Br. 011/009	MLVA31-35
**2**	Apulia	A.Br. 011/009	MLVA31-36
**1**	Apulia	A.Br. 011/009	MLVA31-37
**1**	Lazio	A.Br. 011/009	MLVA31-38
**1**	Lazio	A.Br. 011/009	MLVA31-39
**1**	Tuscany	A.Br. 011/009	MLVA31-40
**1**	Apulia	A.Br. 011/009	MLVA31-41
**1**	Apulia	A.Br. 011/009	MLVA31-42
**1**	Campania	A.Br. 011/009	MLVA31-43
**1**	Abruzzo	A.Br. 011/009	MLVA31-44
**2**	Lazio	A.Br. 011/009	MLVA31-45
**1**	Lazio	A.Br. 011/009	MLVA31-46
**5**	Lazio	A.Br. 011/009	MLVA31-47
**3**	Sicily	A.Br. 008/011	MLVA31-48
**1**	Sicily	A.Br. 008/011	MLVA31-49
**2**	Sicily	A.Br. 008/011	MLVA31-50
**9**	Sicily	A.Br. 008/011	MLVA31-51
**7**	Sicily	A.Br. 008/011	MLVA31-52
**1**	Sicily	A.Br. 008/011	MLVA31-53
**1**	Veneto	A.Br. 005/006	MLVA31-54
**2**	Trentino/Veneto	B.Br. CNEVA	MLVA31-55

Discussion

B. anthracis is considered one of the bacteria with the highest degree of genetic homogeneity and this feature makes difficult to discriminate isolates for epidemiological purpose. This characteristic is motivated by the high survivability of the spores which allow *B. anthracis* to multiply for a relatively limited number of times during its evolution [22].

The 31-loci MLVA analysis performed on 234 *B. anthracis* strains isolated in Italy, during outbreaks occurred from 1972 to 2018, highlights that, until today, 55 genotypes ~~result circulating~~ in Italy. Out of the 55 identified genotypes, 53 belong to a common cluster, and they appear to be genetically very similar each other, supporting the hypothesis that they could be the result of the evolution from a common ancestral strain.

This data is also confirmed by CanSNPs analysis that inserts them in the phylogenetic cluster Trans-Eurasian (TEA).

The analysis of the classical 13 CanSNPs, as a matter of fact, showed that the most of the analyzed strains (98%) belong to the sublineage A.Br.008/009 (Tran Eurasian or TEA group) which is the most represented in Europe and Asia [15]. However, except for the genotypes isolated in Umbria and some others isolated in Sicily belonging to sub-lineage A.Br.008/011, all the others belonged to the sublineage A.Br.011/009.

Interestingly, the genotype GT-54 isolated in Veneto, resulted to be very different from the characteristic Italian strains. This data is confirmed by CanSNPs analysis that inserts the GT54 in Branch A.Br. 005/006, the more ~~diffuse~~ in the central-southern area of Africa, but absent in Europe. Also the genotype GT-55, isolated in Veneto and Trentino is different from most of the Italian strains and belonged to B.Br.CNEVA which is widespread in Europe, in particular, in France, Switzerland and Germany [12, 23, 24].

In Italy, the population of *B. anthracis* is mainly divided into two sublineages A.Br.011/009, definitely the most common, and A.Br.008/011 present only in Umbria and Sicily, both belonging to the large family of TEA (Trans-Eurasian) group (Fig 2).

Fig 2. A UPGMA tree was built using MLVA profiles using Bionumerics 7.6 software (Applied Maths, Belgium). The visualization and the annotation of the phylogenetic tree was performed using the web-based tool Interactive Tree of Life (iTOL). Around the phylogenetic tree, are shown, from the external part to the internal part: genotype number, sublineage, species, year, regions (differently colored) of isolation and identification number of each analyzed strain.

The Trans-Eurasian group A.Br.008/009 rest[illegible] a *B. anthracis* subpopulation well adapted to the Northern hemisphere and predominant all over Europe, Russia, Kazakhstan, Caucasus and the western China [12, 25], and that it led to the Western North American sublineage A.Br.WNA, it is dominant in central part of Canada and much of the western United States. The presence of strains belonging to the A.Br. 008/011 and A.Br. 011/009 sub-lineages could represent the effect of a process of genetic evolution of a common ancestral strain at territorial level. In particular, A.Br. 008/011 represents a rare and deep branching sublineage observed also in Bulgaria, France as well as Turkey [26].

The spread of the TEA group to Europe and Asia seems to be related to the animal handling along the ancient east-west commercial routes of the silk road [27].

Strains belonging to the B.Br.CNEVA lineage are discovered in a relatively small area of the North-Eastern Italy. The relatively low diversity pinpointed between the two strains is consistent with a single introduction of the B.Br.CNEVA lineage into the country, followed by ecological establishment and progressive *in situ* differentiation around the Italian Alps area. Consistent with this hypothesis, the Italian strains form a distinct cluster with respect to other European B.Br. CNEVA - affiliated isolates. The presence of one A.Br.005/006 strain in Italy could be related to trade exchanges dating back to the Maritime Republics period (Middle Ages), when city states

competed for trade and commerce throughout the Mediterranean [7]. This sub-group is well represented in Africa, but rare in Europe [12] and could have been imported through contaminated animal products (e.g. wool, hides, or bone meal) from Africa to Northern Italy. Italy has always been an important trading center in the Mediterranean Sea but also a land of conquest, where several invaders have come and gone over thousands of years.

It is therefore quite surprising that past importations of ill or dying animals or spore-infected items from Africa, the Middle East or even Asia did not impact more the local *B. anthracis* population structure. We assume that exotic genotypes might not have found suitable soils to establish themselves due to the prior presence of strains of the very successful TEA group in geographic areas favorable for *B. anthracis* persistence in most part of the country.

The higher variety of genotypes registered in the regions of Southern Italy, can be also explained by the diversity of breeding systems between Northern and Southern Italy. In Southern Italy there are still many farms rearing animals in the free range, and while they freely graze it can happen, they come across some contaminated fields with anthrax spores. These episodes are less common in Northern Italy, because most of the farms act intensive breeding systems with fixed housing stables.

Another aspect emerging from this study is that just a few genotypes are shared between neighboring regions. In particular the GT-24 is present both in Apulia, Basilicata and Calabria, the GT-26 and GT-27 is shared between Basilicata and Campania and the GT-55 between Veneto and Trentino. Noteworthy and difficult to explain is dislocation of the GT-31 that is shared between Apulia, Tuscany and Sardinia which are not neighboring regions, on the contrary they are quite far from each other.

Most of the genotypes are exclusive for each region, so that it seems that Italian *B. anthracis* strains are autochthonous for single territory. It’s interesting to notice that especially in Sicily and Sardinia islands are present genotypes exclusive just for those regions, probably due to the low number of animal movements between these islands and the rest of Italy.

Definitively, the analysis of these hypervariable regions with methods such as MLVA, is a valuable tool for studying the diversity, evolution, and molecular epidemiology of *B. anthracis*. Moreover, nowadays this assay represents a valid method to understand the distribution of *B. anthracis* within a country.

Acknowledgments

We thank Angela Aceti, Michela Iatarola, Elena Poppa and Francesco Tolve for the technical support.

References

1. Sweeney DA, Hicks CW, Cui X, Li Y, Eichacker PQ. Anthrax infection. Am J Respir Crit Care Med.2011;184:1333- 41. doi:10.1164/rccm.201102-0209CI.
2. Hugh-Jones M, Blackburn J. Theecology of *Bacillus anthracis*. Mol Aspects Med. 2009; 30:356-367.doi:10.1016/j.mam.2009.08.003.
3. Fasanella A, Galante D, Garofolo G, Jones MH. Anthrax undervalued zoonosis. Vet Microbiol. 2010; 140, 318-331.doi:10.1016/j.vetmic.2009.08.016.
4. Manzulli V, Fasanella A, Parisi A, Serrecchia L, Donatiello A, Rondinone V, et al. Evaluation of in vitro antimicrobial susceptibility of *Bacillus anthracis* strains isolated during anthrax outbreaks in Italy from 1984 to 2017. J Vet Sci. 2019;20(1):58-62. doi:10.4142/jvs.2019.20.1.58.
5. Hicks CW, Sweeney DA, Danner RL, Eichacker PQ, Suffredini AF, Feng J, et al. Beneficial effects of stress-dose corticosteroid therapy in canines depend on the severity of staphylococcal pneumonia. Intensive Care Med.2012;38:2063-71. doi:10.1007/s00134-012-2735-5.

6. Inglesby TV, Henderson DA, Barlett JG, Ascher MS, Eitzen E, Friedlander AM, et al. Anthrax as a biological weapon: Medical and public health management. JAMA.1999;281:1735-45. doi:10.1001/jama.281.18.1735.
7. Fasanella A, Van Ert M, Altamura SA, Garofolo G, Buonavoglia C, Leori G, et al. Molecular Diversity of *Bacillus anthracis* in Italy. J Clin Microbiol. 2005; 43(7):3398-401.doi:10.1128/JCM.43.7.3398-3401.2005.
8. PalazzoL, De Carlo E, Santagada G, Serrecchia L, Aceti A, Guarino A, et al. Recent Epidemic-Like Anthrax Outbreaks in Italy: What Are the Probable Causes?. OJVM. 2012; 02(02):74-76. doi:10.4236/ojvm.2012.22012.
9. Fasanella A, Garofolo G, Galante D, Quaranta V, Palazzo L, Lista F, et al. Severe anthrax outbreaks in Italy in 2004: considerations on factors involved in the spread of infection. New Microbiol. 2010;33(1):83-6.
10. Nicastri E, Vairo F, Mencarini P, Battisti A, Agrati C, Cimini E et al. Unexpected human cases of cutaneous anthrax in Latium region, Italy, August 2017: integrated human-animal investigation of epidemiological, clinical, microbiological and ecological factors. Euro Surveill. 2019;24(24). doi: 10.2807/1560-7917.ES.2019.24.24.1800685.
11. Fasanella A, Losito S, Trotta T, Adone R, Massa S, Ciuchini F, Chiocco D. Detection of anthrax vaccine virulence factors by polymerase chain reaction. Vaccine. 2001;19(30):4214-8.doi:10.1016/S0264-410X(01)00159-1.
12. Van Ert MN, Easterday WR, Huynh LY, Okinaka RT, Hugh-Jones ME, Ravel Jacques et al.Global genetic population structure of *Bacillus anthracis*. PLoS One. 2007;2: e461.doi:10.1371/journal.pone.0000461.
13. Marston CK, Allen CA, Beaudry J, Price EP, Wolken SR, Pearson T, et al. Molecular epidemiology of anthrax cases associated with recreational use of animal hides and yarn in the United States. PLoS One. 2011;6(12):e28274. doi:10.1371/journal.pone.0028274.

14. Girault G, Thierry S, Cherchame E, Derzelle S. Application of High-Throughput Sequencing: Discovery of Informative SNPs to Subtype *Bacillus anthracis*. Adv BiosciBiotechnol. 2014;5: 669-677.doi:10.4236/abb.2014.57079.
15. Keim P, Price LB, Klevytska AM, Smith KL, Schupp JM, Okinaka R, et al. Multiple-locus variable-number tandem repeat analysis reveals genetic relationships within *Bacillus anthracis*. J. Bacteriol. 2000; 2928–2936.
16. Keim P, Van Ert MN, Pearson T, Vogler AJ, Huynh LY, Wagner DM. Anthrax molecular epidemiology and forensics: using the appropriate marker for different evolutionary scales. Infect Genet Evol. 2004; 4:205-213.doi:10.1016/j.meegid.2004.02.005.
17. Lista F, Faggioni G, Valjevac S, Ciammaruconi A, Vaissaire J, Le Doujet C, et al. Genotyping of *Bacillus anthracis* strains based on automated capillary 25-loci multiple locus variable-number tandem repeats analysis. BMC Microbiol.2006;6;6:33. doi:10.1186/1471-2180-6-33.
18. Thierry S, Tourterel C, Le FlècheP, Derzelle S, Dekhil N, Mendy C, et al.Genotyping of French *Bacillus anthracis* Strains Based on 31-Loci Multi Locus VNTR Analysis: Epidemiology, Marker Evaluation, and Update of the Internet Genotype Database. PLoS One. 2014;9(6): e95131.doi: 10.1371/journal.pone.0095131.
19. Antwerpen M, Ilin D, Georgieva E, Meyer H, Savov E, Frangoulidis D. MLVA and SNP analysis identified a unique genetic cluster in Bulgarian *Bacillus anthracis* strains. Eur J Clin Microbiol Infect Dis. 2011;30:923-930.
20. Derzelle S, Thierry S.Genetic diversity of *Bacillus anthracis* in Europe: genotyping methods in forensic and epidemiologic investigations. Biosecur Bioterror. 2013;11 Suppl 1:S166-76. doi:10.1089/bsp.2013.0003.
21. Peculi A, Campese E, Serrecchia L, Marino L, Boci J, Bijo B, et al. Genotyping of *Bacillus anthracis* Strains Circulating in Albania. J Bioterror Biodef 2015, 6:1. doi:10.4127/2157-2526.1000131.

22. RumeFI, Affuso A, Serrecchia L, Rondinone V, Manzulli V, Campese E, et al. Genotype Analysis of *Bacillus anthracis* Strains Circulating in Bangladesh. PLoS One. 2016;11(4):e0153548. doi:10.1371/journal.pone.0153548.
23. Derzelle S, Laroche S, Le Flèche P, Hauck Y, Thierry S, Vergnaud G, Madani N. Characterization of genetic diversity of *Bacillus anthracis* in France by using high-resolution melting assays and multi locus variable-number tandem-repeat analysis. J Clin Microbiol.2011;49:4286-4292. doi:10.1128/JCM.05439-11.
24. Steiner I1, Račić I, Spičić S, Habrun B. Genotyping of *Bacillus anthracis* isolated from Croatia and Bosnia and Herzegovina. Zoonoses Public Health. 2013;60(3):202-8. doi:10.1111/j.1863-2378.2012.01513.x.
25. Aikembayev A, Lukhnova L, Temiraliyeva G, Meka-Mechenko T, Pazylov Y, Zakaryan S, et al. Historical Distribution and Molecular Diversity of *Bacillus anthracis*, Kazakhstan. Emerg Infect Dis. 2010; 16(5): 789–796. doi:10.3201/eid1605.091427.
26. Timofeev V, Bahtejeva I, Mironova R, Titareva G, Lev I, Christiany D et al. Insights from *Bacillus anthracis* strains isolated from permafrost in the tundra zone of Russia. PLoS One. 2019;14(5):e0209140. doi: 10.1371/journal.pone.0209140.
27. Simonson TS, Okinaka R., Wang B, Easterday WR, Huynh L, et al. *Bacillus anthracis* in China and its relationship to worldwide lineages. BMC Microbiol. 2009;9: 71. doi:10.1186/1471-2180-9-71.

Fig1

Click here to download Figure Fig1.tif

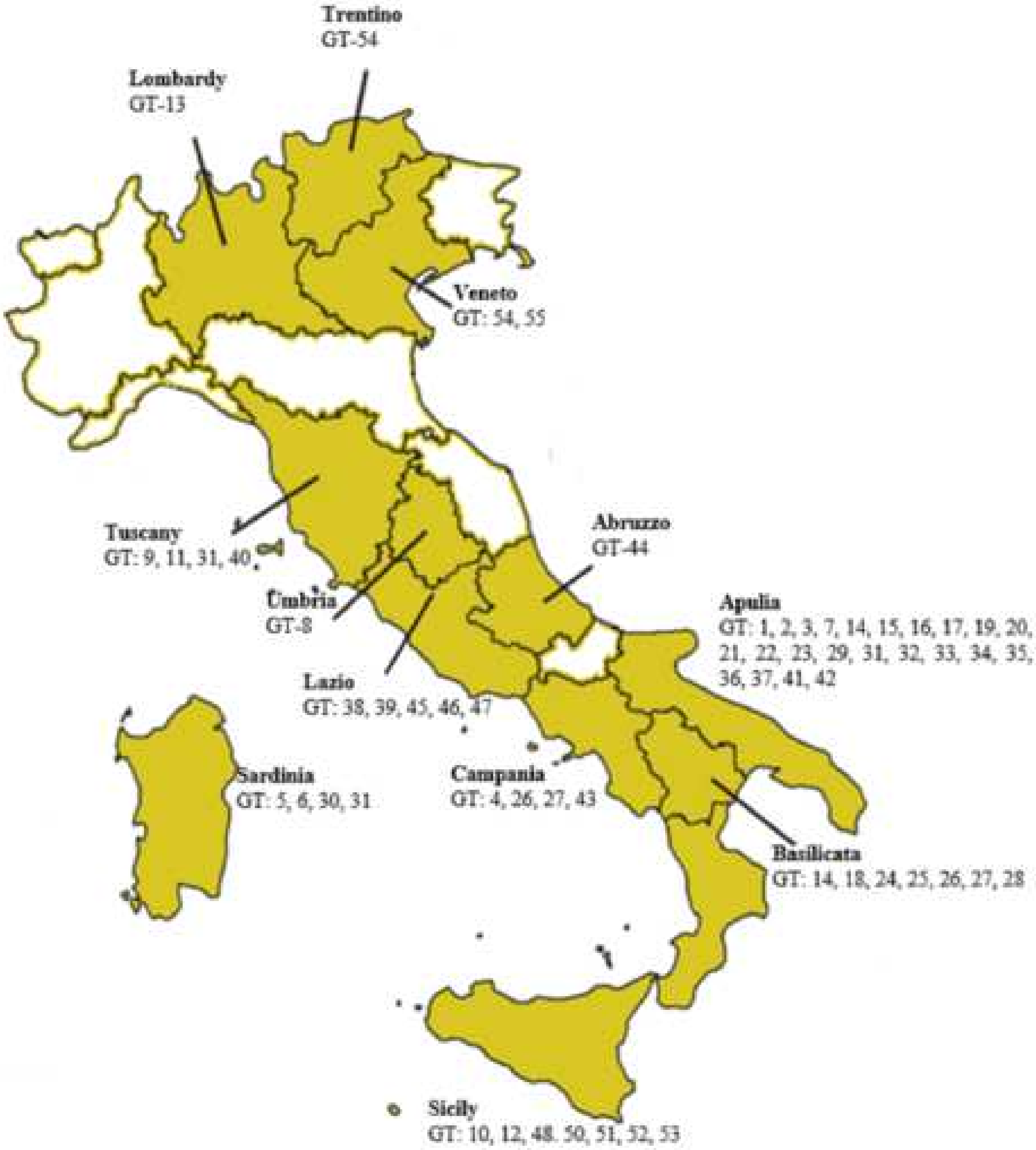

Fig2

Click here to download Figure Fig2.tiff

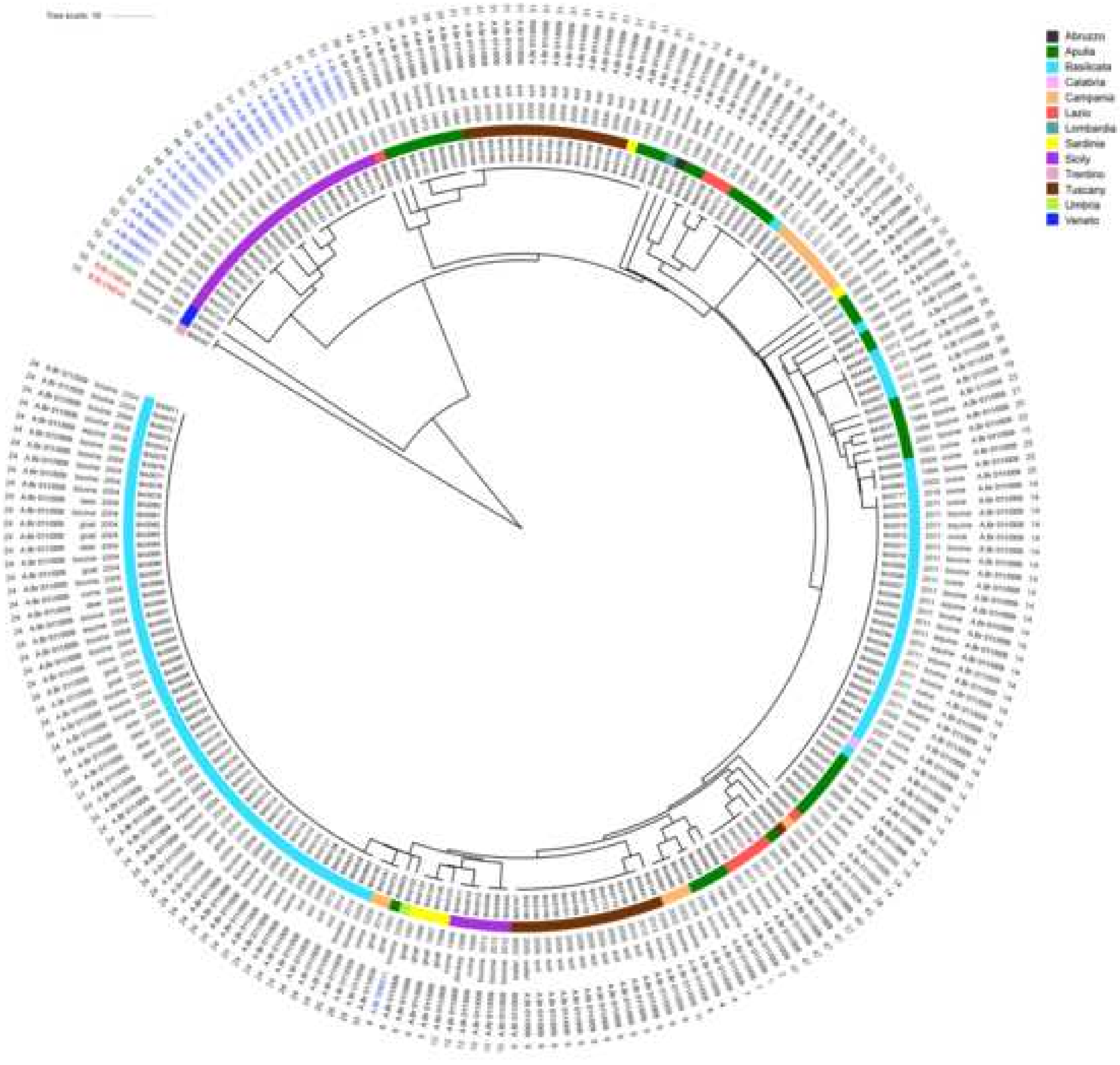

Supplement: Attachment — Submitted filename: PONE-D-19-23489_reviewer (2) reply.pdf [file pone.0227875.s004.pdf]
