# Supplementary material for: Genetic characterization of Bacillus anthracis strains circulating in Italy from 1972 to 2018
Source: PLoS One. 2020 Jan 13;15(1):e0227875. doi: 10.1371/journal.pone.0227875 (PMC6957342; doi:10.1371/journal.pone.0227875)

Manuscript Click here to access/download;Manuscript;Manuscript.docx

Genetic characterization of *Bacillus anthracis* strains circulating in Italy from 1972 to 2018.

Valeria Rondinone [1¶], Luigina Serrecchia[1¶], Antonio Parisi [1¶], Antonio Fasanella [1¶], Viviana Manzulli [1¶], Dora Cipolletta [1¶], Domenico Galante [1*¶]

[1] *Anthrax Reference Institute of Italy, Experimental Zooprophylactic Institute of Apulia and Basilicata Regions, Foggia, Italy*

Short title: Genetic characterization of *Bacillus anthracis* in Italy

**Corresponding author*

E-mail: domenico.galante@izspb.it (DG)

[¶]These authors contributed equally to this work

Abstract

In Italy, anthrax is an endemic disease, with a few outbreaks occurring almost every year. We surveyed 234 *B. anthracis* strains from animals (n=196), humans (n=3) and the environment (n=35) isolated during Italian outbreaks in the years 1972-2018. Despite the considerable genetic homogeneity of *B. anthracis*, the strains were effectively differentiated using canonical single nucleotide polymorphisms (CanSNPs) assay and multiple-locus variable-number tandem repeat analysis (MLVA). The phylogenetic identity was determined through the characterization of 14 CanSNPs. In addition, a subsequent 31-loci MLVA assay was also used to further discriminate *B. anthracis* genotypes into subgroups. The analysis of 14 CanSNPs allowed for the identification of four main lineages: A.Br.011/009, A.Br.008/011 (respectively belonging to A.Br.008/009 sublineage, also known Trans-Eurasian or TEA group), A.Br.005/006 and B.Br.CNEVA. A.Br.011/009, the most common subgroup of lineage A, is the major genotype of *B. anthracis* in Italy. The MLVA analysis, revealed the presence of 55 different genotypes in Italy. Most of the genotypes are genetically very similar, supporting the hypothesis that all strains evolved from a local common ancestral strain, except for two genotypes representing the branch A.Br.005/006 and B.Br.CNEVA. The genotyping analysis applied in this study, remains a very valuable tool for studying the diversity, evolution, and molecular epidemiology of *B. anthracis*.

Introduction

Anthrax is a non-contagious zoonotic disease affecting a broad range of animal species including humans. *Bacillus anthracis*, the etiological agent of anthrax, forms highly resistant spores that can to persist in the environment for several decades [1]. Domestic and wild ruminants are species most susceptible to anthrax [2]. Animals are infected during grazing in areas contaminated with anthrax

spores, while humans can contract the disease by contact with anthrax-infected animals or anthrax-contaminated animal products. Most frequently this involves employment in a specific high risk occupations; such as farmer, butcher, tanner, wool carder, shearer and veterinarian. Exposure most commonly occurs during the skinning and butchering of cattle that are sick or dead because of anthrax [3]. Three forms of anthrax occur in human, depending on exposure type: cutaneous (which is usually non-fatal), gastrointestinal, and inhalational, both of which can be potentially fatal [4]. Recently, a fourth disease form was reported in drug users who inject drugs contaminated with anthrax spores [5]. Further, since it is relatively easy and inexpensive to obtain *B. anthracis*, the bacterium is one of the preferred pathogenic agents for use as bacteriological weapon in bio-terrorist attacks [6]. In Italy, anthrax is typically a sporadic disease, particularly occurring during the summer (with a few exceptions) in the central and southern regions, and in the major islands, where it almost exclusively affects animals at pasture [7]. Between 1972 and 2018, approximately 200 outbreaks of animal anthrax were recorded (unpublished data). Very rarely, anthrax infection takes the form of an epidemic-like disease, characterized by outbreaks in limited areas involving a great number of animals. In Italy, two major epidemic-like anthrax outbreaks have been reported: one during the summer of 2004 in Basilicata, and one during the summer of 2011, in an area between Basilicata and Campania [8, 9]. Molecular tools, such as the canonical SNPs assay (CanSNPs) and multiple-locus variable-number tandem repeat analysis (MLVA), are highly effective for differentiating *B. anthracis* strains. The overall goal of this study was to utilize SNP analysis to establish the phylogenetic relationship between the *B. anthracis* strains examined, and further discriminate them in the context of the MLVA assay, in order to examine correlations among the *B. anthracis* isolates associated with the Italian anthrax outbreaks and to assess genetic diversity at regional and broader scales.

Materials and Methods

Ethics statement

The animal and environmental strains used in the current study were isolated at the Anthrax Reference Institute of Italy (Ce.R.N.A.), a public laboratory, mandated by the Italian Ministry of Health to confirm diagnosis of all animal anthrax cases in Italy. During outbreaks, samples were collected by the veterinary services of the Ministry of Health. Specific permission for soil sampling was not required. *B. anthracis* DNAs from anthrax human cutaneous cases were also included in the current study; two came from the "San Carlo" Hospital, Department of Infectious Disease, Potenza, Italy, and one from the "L. Spallanzani" National Institute for Infectious Disease , Rome, Italy [10].

Bacterial Strains

A collection of 234 *B. anthracis* strains, including 196 strains isolated from animal and 35 from the environment, isolated during Italian anthrax outbreaks in the years 1972-2018, were analyzed in the current study (Table 1). Furthermore, 3 *B. anthracis* DNAs from anthrax human cutaneous cases were also analyzed.

Table 1. Overview of *Bacillus anthracis* isolates from the years 1972-2018 analyzed in the current study.

Sample type	Source	No. of isolates	Regions
Environmental samples	Water	3	Tuscany
	Soil	32	Basilicata, Tuscany
Animal samples	Bovine	101	Basilicata, Campania, Lazio, Apulia, Sardinia, Sicily, Tuscany, Umbria, Veneto, Lombardy
	Caprine	20	Abruzzo, Basilicata, Calabria, Campania, Apulia, Sardinia, Trentino
	Deer	7	Basilicata
	Equine	12	Basilicata, Campania, Apulia
	Ovine	53	Basilicata, Campania, Lazio, Apulia, Sicily
	Swine	3	Basilicata

Human samples (DNAs)	Human	3	Basilicata, Lazio

DNA Extraction

B. anthracis strains were seeded on 5% sheep blood agar plates and then incubated at +37°C for 24 h. Bacterial DNA was extracted using the DNAeasy Blood and Tissue kit (Qiagen, Hilden, Germany), following the protocol for Gram-positive bacteria. All manipulations of *B. anthracis* strains were performed in a biosafety level 3 laboratory at the Experimental Zooprophylactic Institute of Apulia and Basilicata Regions in a class II type A 2 biosafety cabinet.

Real-time polymerase chain reaction (PCR) assay

Molecular identification of *B. anthracis* was performed using qualitative real-time PCR. The method is based on the amplification of specific DNA sequences using three pairs of specific primers [11] as follows: R1/R2 primers, specific for the BA813 gene located on the *B. anthracis* chromosome; PAG 23/24 primers, specific for the protective antigen (PA) gene located on the virulence plasmid pXO1; and CAP 57/58 primers, specific for the capsule (CAP) gene located on the virulence plasmid pXO2. Each 20 µl reaction mixture contained 1x Sso Advanced TM SYBR® Green Supermix (BIORAD), 300 nM each forward and reverse primer, and approximately 10 ng DNA template. The amplification was performed using the CFX Connect Real Time PCR Detection System (BIORAD). A melting curve was generated at 0.5°C increments between 65°C and 95°C, and was analyzed by CFX Manager TM Software, Version 3.0 (BIORAD).

CanSNP analysis

CanSNP profiles were obtained using 13 allelic discrimination assays involving specific oligonucleotides and probes, as described by Van Ert et al. [12]. Each 10 µl reaction mixture contained 1x TaqMan Genotyping Master Mix (Applied Biosystems, Foster City, CA, USA), 250 nM

probe, 600 nM each of forward and reverse primer, and approximately 10 ng DNA template. For all assays, the thermal cycling parameters used were as follows: 10 min at 95°C, followed by 40 cycles of 15 s at 95°C and 1 min at 60°C. Endpoint fluorescent data were acquired by using the ABI 7900HT instrument. The CanSNPs data were compared with the data for 12 recognized sublineage or subgroups. The 14th SNP was detected a High Resolution Melting (HRM) assay for a specific A.Br.011 CanSNP [13,14]. Position 2,552,486, based on the Ames Ancestor genome (NC_007530.2), was analyzed. Amplification was performed using the CFX Connect Real Time PCR Detection System (BIORAD) and Precision Melt Supermix (BIORAD). The reaction mixture contained 0.2 μM of each primer and 1x Precision Melt Supermix (BIORAD) in a 20 μl final volume. The following cycling parameters were used: 2 min at 95°C, were followed by 35 cycles of 10 s at 95°C and 30 s at 60°C. The samples were then heated to 95°C for 30 s, cooled down to 60°C over 1 min, and then heated from 65°C to 95°C at a rate of 0.5°C/s. High-resolution melting data were analyzed using Precision Melt Analysis Software (BIORAD).

31-loci MLVA analysis

For the 31-marker MLVA, 5' fluorescently labeled oligonucleotides (6-FAM, VIC, NED and PET), specifically selected for variable number tandem repeats (VNTR) analysis, we used. Twenty-seven chromosomal VNTR loci (vrrA, vrrB1, cg3, vrrB2, vntr19, vrrC1, vrrC2, vntr32, vntr12, vntr35, vntr23, bams03, bams05, bams13, bams15, bams21, bams22, bams23, bams24, bams25, bams28, bams30, bams31, bams34, bams44, bams51 and bams53) and four plasmid loci (vntr16, vntr17, pxO1 and pxO2) [12, 15-18] were analyzed. The MLVA assay involved preparation of two singleplex and nine multiplex reactions, in a final volume of 15 μl. Each reaction mixture contained the following: 1x PCR reaction buffer (Qiagen, Hilden, Germany); 3 mM $MgCl_2$, 0.2 mM for each dNTP; 1 U Hot Star Plus Taq DNA polymerase (Qiagen, Hilden, Germany), the appropriate concentration of each primer (as described in Table 2); and approximately 10 ng DNA template .

Table 2. Primer concentration for each set of marker in PCR reactions of MLVA analysis.

PCR Reactions	Primers conc. [μM]
Singleplex 1	vrrC1 [0.2 μM]
Singleplex 2	vrrC2 [0.2 μM]
Multiplex 1	vrrA, vrrB1 [0.2 μM]; CG3 [0.4 μM]
Multiplex 2	vrrB2 [0.25 μM]; pXO1 [0.3 μM]; pXO2 [0.1 μM]
Multiplex 3	vntr12 [0.25 μM]; vntr19 [0.2 μM]; vntr35 [0.2 μM]
Multiplex 4	vntr16 [0.25 μM]; vntr23 [0.2 μM]
Multiplex 5	vntr17 [0.1 μM]; vntr32 [0.4 μM]
Multiplex 6	bams03 [0.8 μM]; bams05 [0.2 μM]; bams15, bams44 [0.5 μM]
Multiplex 7	bams21 [0.4 μM]; bams24, bams25 [0.3 μM]; bams34 [0.2 μM]
Multiplex 8	bams13 [0.3 μM]; bams28 [0.15 μM]; bams31, bams53 [0.6 μM]
Multiplex 9	bams22, bams51 [0.3 μM]; bams23 [0.2 μM]; bams30 [0.6 μM]

The following PCR cycling program was used for the two singleplex reactions and for multiplex reactions 1 and 2: 5 min at 95°C; followed by 35 cycles of 30 s at 94°C, 30 s at 60°C, and 30 s at 72°C, with a final step of 5 min at 72°C. The following amplification program was used for multiplex reactions 3: 5 min at 95°C, followed by 35 cycles of 30 s at 94°C, 30 s at 54°C, 45 s at 72°C, and 5 min at 72°C. The following amplification program was used for multiplex reaction 4: 5 min at 95°C, followed by 35 cycles of 30 s at 94°C, 45 s at 56°C, 1 min at 72°C, and 5 min at 72°. The following amplification program was used for multiplex reaction 5: 5 min at 95°C , followed by 35 cycles of 30 s at 94°C, 45 s at 59°, 1 min at 72°C, and 5 min at 72°C. The following amplification program was used for multiplex reactions 6 to 9: 5 min at 94°C, followed by 35 cycles of 1 min at 94°C, 90 s at 60°, 90 s at 72°C, and 15 min at 72°C.

Automated genotype analysis

The MLVA PCR products were diluted 1:80 and analyzed by capillary electrophoresis using the ABI Prism 3130 genetic analyzer (Applied Biosystems) and 0.25 µl GeneScan 1200, and were sized by using Gene Mapper 4.0 (Applied Biosystems Inc.). Assignment of the sizes and corresponding repeating unit numbers for each locus was performed using the following strains as references: Ames Ancestor (NC_007530.2, chromosome), pXO1 (Nc_007322.2, plasmid), and pXO2 (NC_007323.2, plasmid). Data were analyzed using conventional values proposed in the updated version of the 2016 *Bacillus anthracis* MLVA database, available at MLVAbank (http://mlva.u-psud.fr/). Phylogram was derived by clustering with the unweighted pair group method with arithmetic means (UPGMA), using 'categorical' character table values. All markers were given equal weight, irrespective of the number of repeats.

The discriminatory ability of the MLVA technique was determined by calculating the discriminatory index (D) for 234 typed strains. The discriminatory power of each VNTR was estimated by the number of alleles detected and the allele diversity using BioNumerics 7.6 software (Applied Maths, Belgium) [19].

Results

Real Time PCR, CanSNPs and MLVA analysis of anthrax strains

All the analyzed strains tested positive after the PCR amplification of chromosomal, plasmid pXO1 (toxins coding) and pXO2 (capsule formation) targets.

The analysis of 13 classical CanSNPs, revealed that 231 analyzed strains belonged to the sublineage A.Br. 008/009, or also known as Trans-Eurasian (TEA) group. The TEA group was established in southern and eastern Europe, and represents the dominant subgroup in Italy, Bulgaria, Hungary and Albania [7, 12, 20-22]. The analysis of an additional, 14th, CanSNP (A.Br.011), recently allowed to division of the A.Br.008/009 group into 2 subgroups. Accordingly, the analysis of the 14th CanSNP in the current study revealed that 207 of the 231 strains belonged to the main sub-lineage

awkward wording.

recently enabled division of the A.Br.008/009 group into 2 subgroups &

OR recently allowed for the differentiation of the .... etc.

A.Br.011/009, while 24 strains belonged to the sublineage A.Br.008/011. All but one strain belonging to the latter sublineage were isolated in Sicily; one strain was isolated in Umbria. Further, one strain isolated in Veneto belonged to the main lineage A, sublineage A.Br.005/006, while two other strains, one from Veneto and one from Trentino, belonged to the main lineage B, sublineage B.Br.CNEVA.

MLVA based on the analysis of 31 VNTRs, revealed 55 different genotypes, as shown in S1 table, distributed in the Italian regions, named GT-1 to GT-55, accordingly (Fig 1). The GT-14 genotype was the most common and was represented by 34 strains, mostly from Basilicata, Apulia, and Calabria. The GT-31 genotype was represented by 19 isolates: 16 from Tuscany, two from Apulia and one from Sardinia. The GT-26 and GT-27 genotypes were only isolated in the Basilicata and Campania regions. Other genotypes were characteristic for single regions, as showed in Table 3.

Table 3. Distribution of *Bacillus anthracis* CanSNPs and genotypes isolated in Italy in the years 1972-2018.

Number of isolates	Regions	CanSNPs sublineage	Genotype
1	Apulia	A.Br. 011/009	MLVA31-1
1	Apulia	A.Br. 011/009	MLVA31-2
1	Apulia	A.Br. 011/009	MLVA31-3
3	Campania	A.Br. 011/009	MLVA31-4
1	Sardinia	A.Br. 011/009	MLVA31-5
3	Sardinia	A.Br. 011/009	MLVA31-6
2	Apulia	A.Br. 011/009	MLVA31-7
1	Umbria	A.Br. 008/011	MLVA31-8
14	Tuscany	A.Br. 011/009	MLVA31-9
3	Sicily	A.Br. 011/009	MLVA31-10
1	Tuscany	A.Br. 011/009	MLVA31-11
3	Sicily	A.Br. 011/009	MLVA31-12
1	Lombardy	A.Br. 011/009	MLVA31-13
34	Basilicata/Apulia/Calabria	A.Br. 011/009	MLVA31-14

1	Apulia	A.Br. 011/009	MLVA31-15
2	Apulia	A.Br. 011/009	MLVA31-16
1	Apulia	A.Br. 011/009	MLVA31-17
1	Basilicata	A.Br. 011/009	MLVA31-18
1	Apulia	A.Br. 011/009	MLVA31-19
1	Apulia	A.Br. 011/009	MLVA31-20
1	Apulia	A.Br. 011/009	MLVA31-21
1	Apulia	A.Br. 011/009	MLVA31-22
1	Apulia	A.Br. 011/009	MLVA31-23
57	Basilicata	A.Br. 011/009	MLVA31-24
3	Basilicata	A.Br. 011/009	MLVA31-25
3	Campania/Basilicata	A.Br. 011/009	MLVA31-26
9	Campania/Basilicata	A.Br. 011/009	MLVA31-27
5	Basilicata	A.Br. 011/009	MLVA31-28
1	Apulia	A.Br. 011/009	MLVA31-29
1	Sardinia	A.Br. 011/009	MLVA31-30
19	Tuscany/Apulia/Sardinia	A.Br. 011/009	MLVA31-31
1	Apulia	A.Br. 011/009	MLVA31-32
1	Apulia	A.Br. 011/009	MLVA31-33
5	Apulia	A.Br. 011/009	MLVA31-34
6	Apulia	A.Br. 011/009	MLVA31-35
2	Apulia	A.Br. 011/009	MLVA31-36
1	Apulia	A.Br. 011/009	MLVA31-37
1	Lazio	A.Br. 011/009	MLVA31-38
1	Lazio	A.Br. 011/009	MLVA31-39
1	Tuscany	A.Br. 011/009	MLVA31-40
1	Apulia	A.Br. 011/009	MLVA31-41
1	Apulia	A.Br. 011/009	MLVA31-42
1	Campania	A.Br. 011/009	MLVA31-43
1	Abruzzo	A.Br. 011/009	MLVA31-44
2	Lazio	A.Br. 011/009	MLVA31-45
1	Lazio	A.Br. 011/009	MLVA31-46
5	Lazio	A.Br. 011/009	MLVA31-47

3	Sicily	A.Br. 008/011	MLVA31-48
1	Sicily	A.Br. 008/011	MLVA31-49
2	Sicily	A.Br. 008/011	MLVA31-50
9	Sicily	A.Br. 008/011	MLVA31-51
7	Sicily	A.Br. 008/011	MLVA31-52
1	Sicily	A.Br. 008/011	MLVA31-53
1	Veneto	A.Br. 005/006	MLVA31-54
2	Trentino/Veneto	B.Br. CNEVA	MLVA31-55

The number of different alleles ranged from 1 for bams21 and bams25 to 10 for bams15. Highest allelic diversities measured by Simpson's discriminatory index (0.531047) was observed for the locus pXO1aat (Table 4). The relationship among the strains based on MLVA results is represented in Figure 2.

Table 4. Simpson's Index of Diversity and allele numbers of MLVA markers with respect to the collection investigated.

Locus	No. alleles	Diversity Index (Simpson)
vrrA	4	0.799876
vrrB1	2	0.983052
vrrB2	3	0.933523
vrrC1	2	0.983052
vrrC2	2	0.910403
CG3	2	0.974688
pXO1aat	4	0.531047
pXO2at	4	0.88593
vntr32	3	0.974615
bams03	2	0.983052
bams05	5	0.924252
bams13	5	0.848418
bams15	10	0.590693

Reviewer 1: Observation

I am more accustomed to the Shanon diversity index for allelic diversity calculations.

Your sample size is sufficient for it.

Shanon is better at representing diversity & species richness.

It also makes your dataset comparable to Thierry et al 2014, Van Ert etc.

bams21	1	1
bams22	3	0.901965
bams23	4	0.949704
bams24	4	0.763642
bams25	1	1
bams28	2	0.640405
bams30	6	0.908759
bams31	7	0.7685
bams34	3	0.974363
bams44	2	0.809153
bams51	5	0.82391
bams53	3	0.982834
vntr12	4	0.925086
vntr16	5	0.739828
vntr17	4	0.724049
vntr19	2	0.64497
vntr23	2	0.926036
vntr35	2	0.78932

Fig 1. The geographical distribution of 55 *Bacillus anthracis* genotypes in Italy. Image modified from the "Map of Italy"; "World of Maps" Public Domain (*https://www.worldofmaps.net/europa/landkarten-und-stadtplaene-von-italien/landkarte-italien-administrative-bezirke-regioni.htm*)

Fig 2. A UPGMA phylogram of MLVA profiles. The phylogram was built using BioNumerics 7.6 software (Applied Maths, Belgium). The visualization and the annotation of the genetic distances were performed using the web-based tool Interactive Tree of Life (iTOL). Around the phylogram are shown, from the external part to the internal part: genotype number, sublineage, species, year, regions (differently colored) of isolation and identification number of each analyzed strain.

Discussion

Bacillus (starting a sentence: write out in full)

B. anthracis is clonal in nature, often exhibits a high degree of genetic homogeneity. This characteristic has traditionally made the discrimination of isolates for epidemiological purpose difficult. This may be explained by the high survivability of spores, which allowed *B. anthracis* to reproduce a relatively limited number of times during its evolution [23]. The 31-loci MLVA analysis of 234 *B. anthracis* strains isolated in Italy during outbreaks in the years 1972-2018 reported herein, revealed that, to date, 55 *B. anthracis* genotypes circulate in Italy. The performed CanSNPs analysis placed 53 of the 55 identified genotypes in a common cluster (TEA). The analysis of the classical 13 CanSNPs, revealed that most of the analyzed strains (98%) belonged to the sublineage A.Br.008/009 (the TEA group), which is the most common group in Europe and Asia [15]. However, except for the genotypes of strains isolated in Umbria and some others isolated in Sicily belonging to sublineage A.Br.008/011, all strains belonged to the sublineage A.Br.011/009. Interestingly, genotype GT-54, isolated in Veneto, was very different from the other characteristic Italian strains. CanSNPs analysis confirmed this observation, placing this genotype in the branch A.Br.005/006. This branch is generally present in the central-southern Africa, although it was also identified in Europe [12, 24]. Furthermore, genotype GT-55, isolated in Veneto and Trentino, is different from those of most Italian strains, and it belonged to B.Br.CNEVA. This branch is mainly widespread in Europe, in particular in France, Switzerland and Germany [12, 25, 26]. In Italy, the population of *B. anthracis* is mainly divided into two sublineages: A.Br.011/009, definitely the most common, and A.Br.008/011 present only in Umbria and Sicily. Both these sublineages belong to the large TEA group (Fig 2). The TEA group A.Br.008/009 contains a *B. anthracis* subpopulation that is well adapted to the northern hemisphere and predominant in Europe, Russia, Kazakhstan, Caucasus and western China [12, 27]. It has also been detected in Africa [18, 28]. This group gave rise to the western north American sublineage (A.Br.WNA), which is

dominant in central Canada and much of the western USA. The presence of strains belonging to sublineages A.Br.008/011 and A.Br.011/009 might represent an effect of genetic evolution of a common ancestral strain at territorial level. In particular, A.Br.008/011 represents a rare and deep branching sublineage, also observed in Bulgaria, France as well as Turkey [29]. The spread of the TEA group to Europe and Asia seems to be linked to animal handling along the ancient East-West commercial routes of the Silk Road [30]. In the current study, strains belonging to the B.Br.CNEVA lineage were isolated in a relatively small area of north-eastern Italy. The relatively low diversity between the two strains demonstrated in the current study is consistent with a single introduction event of the B.Br.CNEVA lineage into the country, followed by ecological establishment and progressive *in situ* differentiation around the Italian Alps area [21]. Consistent with this hypothesis, the Italian strains form a cluster that is distinct from the other European B.Br.CNEVA. The identification of one A.Br.005/006 strain in Italy could be associated with the trade exchanges dating back to the Maritime Republics period (the Middle Ages), when city states competed for trade and commerce throughout the Mediterranean [7]. This subgroup is well represented in Africa, but rare in Europe [12]. It is therefore quite surprising that past importations of ill or dying animals or spore-infected items from Africa, the Middle East, or even Asia, did not have a greater impact on the genetic structure of *B. anthracis* in the region. The higher variety of *B. anthracis* genotypes identified in southern Italy relative to genotypes from other Italian regions, can be explained by the differences in the breeding systems between northern and southern Italy. In southern Italy, many livestock farmers use extensive farming methods, increasing the chances of grazer exposure to historical spore sites and deposits. This possibility of exposure is lower in northern Italy because most livestock farmers use intensive breeding systems. Another observation from the current study was that the neighboring regions share just a few genotypes. In particular, the GT-24 genotype was present in Apulia, Basilicata and Calabria; the GT-26 and GT-27 genotypes were identified in Basilicata and Campania; and the GT-55 genotype was identified in Veneto and Trentino. Noteworthy and difficult to explain is the dislocation of genotype GT-31, identified in Apulia,

insufficient evidence in this study to support this hypothesis. A.Br.005/006 is present in Europe & this study → 1970s – 2018 limit.

Tuscany, and Sardinia. ~~This~~ These are not neighboring regions; on the contrary, they are quite far from one another .Also in this national scenario one of the explanations could be the trade of animals or animal products within the country over the years. Nevertheless, since most genotypes are exclusive to each region, it appears that Italian *B. anthracis* strains may be autochthonous for a single territory. Interestingly, genotypes exclusive to specific regions were detected especially in Sicily and Sardinia, probably because of low animal movements between these islands and the rest of Italy. The analysis of chromosomal and plasmid hypervariable regions using such methods as MLVA constitutes a valuable approach for studying the diversity, evolution and molecular epidemiology of *B. anthracis*. Therefore, MLVA is a valid method that enables the understanding of the distribution of *B. anthracis* within a country.

Acknowledgments

We thank Angela Aceti, Michela Iatarola, Elena Poppa and Francesco Tolve for the technical support.

Author Contributions

Conceptualization: Valeria Rondinone, Domenico Galante, Luigina Serrecchia, Antonio Fasanella

Data curation: Valeria Rondinone, Luigina Serrecchia, Antonio Parisi, Viviana Manzulli, Dora Cipolletta

Formal analysis: Luigina Serrecchia, Antonio Parisi

Funding acquisition: Antonio Fasanella

Investigation: Valeria Rondinone, Domenico Galante, Luigina Serrecchia, Viviana Manzulli

Methodology: Domenico Galante, Luigina Serrecchia, Antonio Parisi

Project administration: Antonio Fasanella

Resources: Antonio Fasanella

Supervision: Domenico Galante, Antonio Parisi, Antonio Fasanella

Validation: Luigina Serrecchia, Antonio Parisi

Visualization: Valeria Rondinone, Dora Cipolletta

Writing – original draft: Valeria Rondinone, Domenico Galante, Luigina Serrecchia, Viviana Manzulli, Dora Cipolletta

Writing – review & editing: Valeria Rondinone, Domenico Galante, Luigina Serrecchia, Antonio Parisi, Viviana Manzulli, Antonio Fasanella, Dora Cipolletta

References

1. Dragon DC, Rennie RP (1995) The ecology of anthrax spores: tough but not invincible. Can Vet J. 36: 295-301.
2. Hugh-Jones M, Blackburn J. The ecology of *Bacillus anthracis*. Mol Aspects Med. 2009; 30:356-367.doi:10.1016/j.mam.2009.08.003.
3. Fasanella A, Galante D, Garofolo G, Jones MH. Anthrax undervalued zoonosis. Vet Microbiol. 2010; 140, 318-331.doi:10.1016/j.vetmic.2009.08.016.
4. Manzulli V, Fasanella A, Parisi A, Serrecchia L, Donatiello A, Rondinone V, et al. Evaluation of in vitro antimicrobial susceptibility of *Bacillus anthracis* strains isolated during anthrax outbreaks in Italy from 1984 to 2017. J Vet Sci. 2019;20(1):58-62. doi:10.4142/jvs.2019.20.1.58.
5. Hicks CW, Sweeney DA, Cui X, Li Y, Eichacker PQ. An overview of anthrax infection including the recently identified form of disease in injection drug users. Intensive Care Med. 2012;38(7):1092-104. doi:10.1007/s00134-012-2541-0.
6. Inglesby TV, Henderson DA, Barlett JG, Ascher MS, Eitzen E, Friedlander AM, et al. Anthrax as a biological weapon: Medical and public health management. JAMA.1999;281:1735-45. doi:10.1001/jama.281.18.1735.

7. Fasanella A, Van Ert M, Altamura SA, Garofolo G, Buonavoglia C, Leori G, et al. Molecular Diversity of *Bacillus anthracis* in Italy. J Clin Microbiol. 2005; 43(7):3398-401. doi:10.1128/JCM.43.7.3398-3401.2005.
8. Palazzo L, De Carlo E, Santagada G, Serrecchia L, Aceti A, Guarino A, et al. Recent Epidemic-Like Anthrax Outbreaks in Italy: What Are the Probable Causes?. OJVM. 2012; 02(02):74-76. doi:10.4236/ojvm.2012.22012.
9. Fasanella A, Garofolo G, Galante D, Quaranta V, Palazzo L, Lista F, et al. Severe anthrax outbreaks in Italy in 2004: considerations on factors involved in the spread of infection. New Microbiol. 2010;33(1):83-6.
10. Nicastri E, Vairo F, Mencarini P, Battisti A, Agrati C, Cimini E et al. Unexpected human cases of cutaneous anthrax in Latium region, Italy, August 2017: integrated human-animal investigation of epidemiological, clinical, microbiological and ecological factors. Euro Surveill. 2019;24(24). doi:10.2807/1560-7917.ES.2019.24.24.1800685.
11. Ramisse V, Patra G, Garrigue H, Guesdon JL, Mock M. Identification and characterization of *Bacillus anthracis* by multiplex PCR analysis of sequences on plasmids pXO1 and pXO2 and chromosomal DNA. FEMS 1996;145:9–16. doi:10.1111/j.1574-6968.1996.tb08548.x .
12. Van Ert MN, Easterday WR, Huynh LY, Okinaka RT, Hugh-Jones ME, Ravel Jacques et al. Global genetic population structure of *Bacillus anthracis*. PLoS One. 2007;2:e461. doi:10.1371/journal.pone.0000461.
13. Marston CK, Allen CA, Beaudry J, Price EP, Wolken SR, Pearson T, et al. Molecular epidemiology of anthrax cases associated with recreational use of animal hides and yarn in the United States. PLoS One. 2011;6(12):e28274. doi:10.1371/journal.pone.0028274.
14. Girault G, Thierry S, Cherchame E, Derzelle S. Application of High-Throughput Sequencing: Discovery of Informative SNPs to Subtype *Bacillus anthracis*. Adv BiosciBiotechnol. 2014;5: 669-677. doi:10.4236/abb.2014.57079.

15. Keim P, Price LB, Klevytska AM, Smith KL, Schupp JM, Okinaka R, et al. Multiple-locus variable-number tandem repeat analysis reveals genetic relationships within *Bacillus anthracis*. J Bacteriol. 2000; 182(10):2928-36. doi: 10.1128/jb.182.10.2928-2936.2000.

16. Keim P, Van Ert MN, Pearson T, Vogler AJ, Huynh LY, Wagner DM. Anthrax molecular epidemiology and forensics: using the appropriate marker for different evolutionary scales. Infect Genet Evol. 2004; 4:205-213. doi:10.1016/j.meegid.2004.02.005.

17. Lista F, Faggioni G, Valjevac S, Ciammaruconi A, Vaissaire J, Le Doujet C, et al. Genotyping of *Bacillus anthracis* strains based on automated capillary 25-loci multiple locus variable-number tandem repeats analysis. BMC Microbiol.2006;6;6:33. doi:10.1186/1471-2180-6-33.

18. Thierry S, Tourterel C, Le FlècheP, Derzelle S, Dekhil N, Mendy C, et al. Genotyping of French *Bacillus anthracis* Strains Based on 31-Loci Multi Locus VNTR Analysis: Epidemiology, Marker Evaluation, and Update of the Internet Genotype Database. PLoS One. 2014;9(6): e95131. doi:10.1371/journal.pone.0095131.

19. Hunter PR, Gaston MA. Numerical index of the discriminatory ability of typing systems: an application of Simpson's index of diversity. J Clin Microbiol. 1988; 26(11):2465-6.

20. Antwerpen M, Ilin D, Georgieva E, Meyer H, Savov E, Frangoulidis D. MLVA and SNP analysis identified a unique genetic cluster in Bulgarian *Bacillus anthracis* strains. Eur J Clin Microbiol Infect Dis. 2011;30:923-930. doi:10.1007/s10096-011-1177-2.

21. Derzelle S, Thierry S. Genetic diversity of *Bacillus anthracis* in Europe: genotyping methods in forensic and epidemiologic investigations. Biosecur Bioterror 2013;11 Suppl 1:S166-76. doi:10.1089/bsp.2013.0003.

22. Peculi A, Campese E, Serrecchia L, Marino L, Boci J, Bijo B, et al. Genotyping of *Bacillus anthracis* Strains Circulating in Albania. J Bioterror Biodef 2015, 6:1. doi:10.4127/2157-2526.1000131.

23. Rume FI, Affuso A, Serrecchia L, Rondinone V, Manzulli V, Campese E, et al. Genotype Analysis of *Bacillus anthracis* Strains Circulating in Bangladesh. PLoS One. 2016;11(4):e0153548. doi:10.1371/journal.pone.0153548.
24. Derzelle S, Girault G, Kokotovic B, Angen Ø. Whole Genome-Sequencing and Phylogenetic Analysis of a Historical Collection of *Bacillus anthracis* Strains from Danish Cattle. PLoS One. 2015;10(8):e0134699. doi: 10.1371/journal.pone.0134699.
25. Derzelle S, Laroche S, Le Flèche P, Hauck Y, Thierry S, Vergnaud G, et al.Characterization of genetic diversity of *Bacillus anthracis* in France by using high-resolution melting assays and multi locus variable-number tandem-repeat analysis. J Clin Microbiol.2011;49:4286-4292. doi:10.1128/JCM.05439-11.
26. Steiner I1, Račić I, Spičić S, Habrun B. Genotyping of *Bacillus anthracis* isolated from Croatia and Bosnia and Herzegovina. Zoonoses Public Health. 2013;60(3):202-8. doi:10.1111/j.1863-2378.2012.01513.x.
27. Aikembayev A, Lukhnova L, Temiraliyeva G, Meka-Mechenko T, Pazylov Y, Zakaryan S, et al. Historical Distribution and Molecular Diversity of *Bacillus anthracis*, Kazakhstan. Emerg Infect Dis. 2010; 16(5): 789–796. doi:10.3201/eid1605.091427.
28. Beyer W, Bellan S, Eberle G, Ganz HH, Getz WM, Haumacher R, et al. Distribution and molecular evolution of *Bacillus anthracis* genotypes in Namibia. PLoS Negl Trop Dis. 2012;6(3):e1534. doi:10.1371/journal.pntd.0001534.
29. Timofeev V, Bahtejeva I, Mironova R, Titareva G, Lev I, Christiany D et al. Insights from *Bacillus anthracis* strains isolated from permafrost in the tundra zone of Russia. PLoS One. 2019;14(5):e0209140. doi:10.1371/journal.pone.0209140.
30. Simonson TS, Okinaka R., Wang B, Easterday WR, Huynh L, et al. *Bacillus anthracis* in China and its relationship to worldwide lineages. BMC Microbiol. 2009;9:71. doi:10.1186/1471-2180-9-71.

Supporting information

S1 Table. Allele distribution of the 55 genotypes identified using 31 VNTR analysis (xlxs).

Click here to access/download;Figure;Fig1.tif

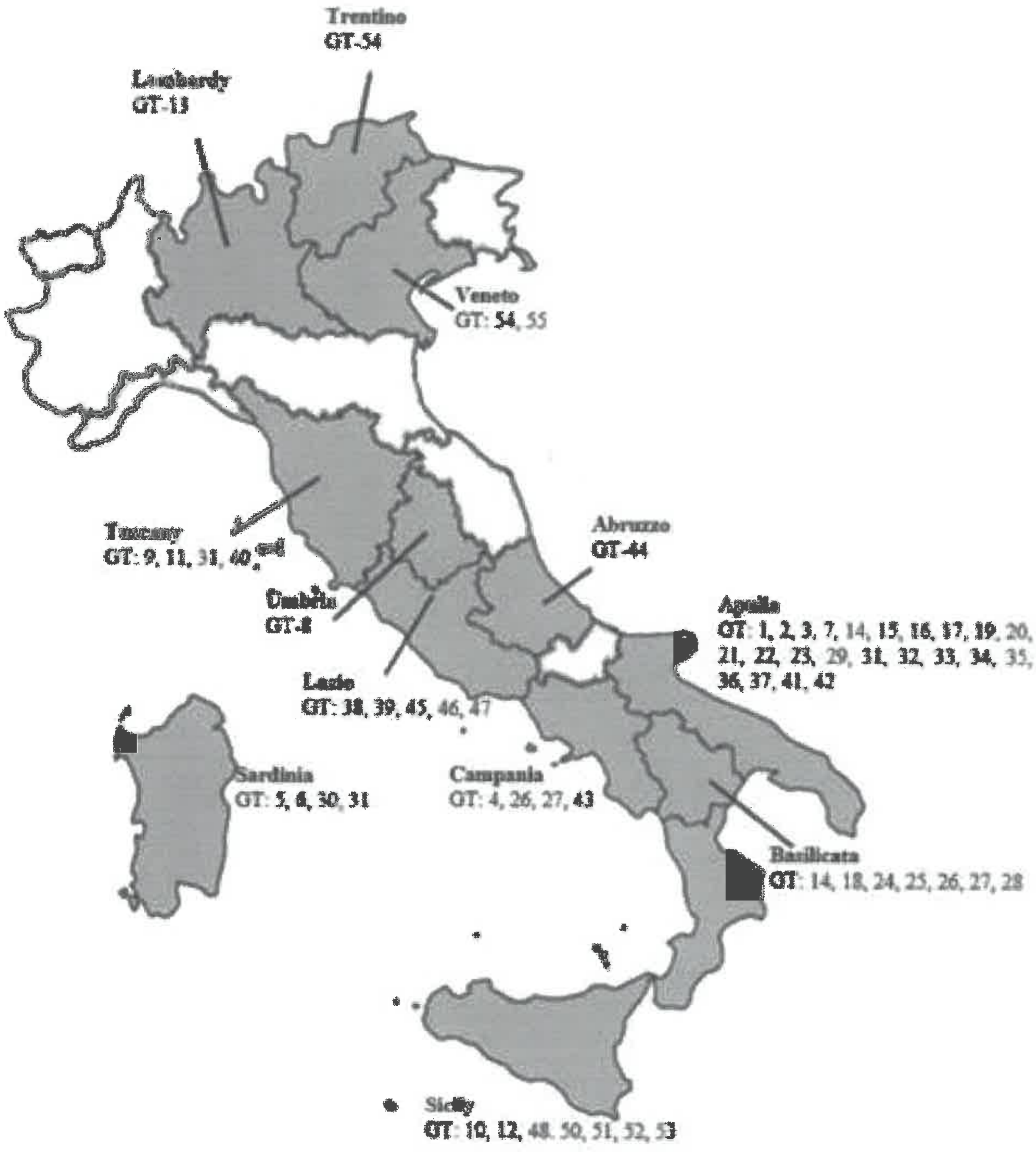

Click here to access/download;Figure;Fig2.tif

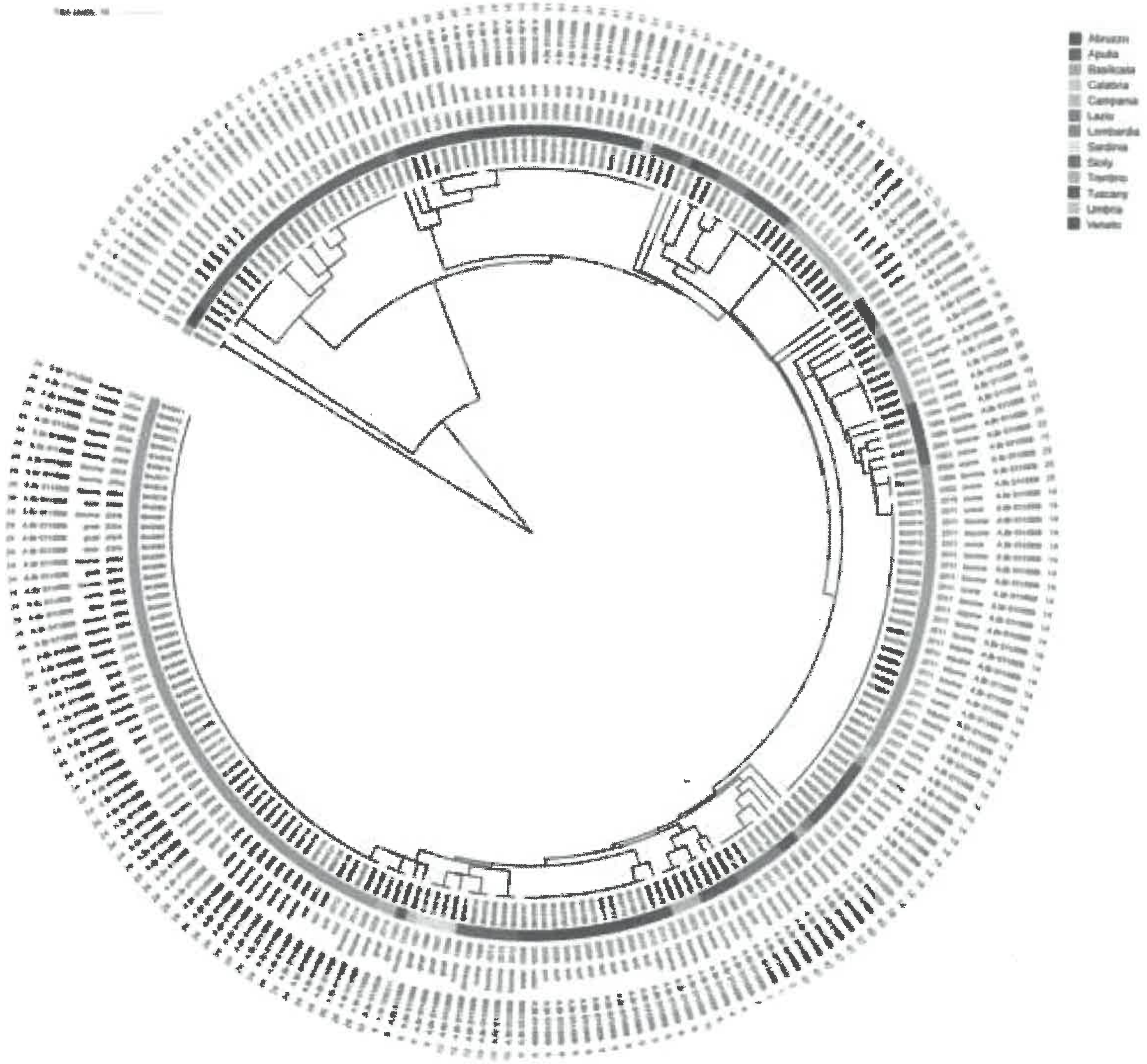

Supplement: Attachment — Submitted filename: PONE-D-19-23489_Reviewer1_02122019.pdf [file pone.0227875.s005.pdf]
