# Supplementary material for: Genetic characterization of Bacillus anthracis strains circulating in Italy from 1972 to 2018
Source: PLoS One. 2020 Jan 13;15(1):e0227875. doi: 10.1371/journal.pone.0227875 (PMC6957342; doi:10.1371/journal.pone.0227875)
Supplement: Attachment — Submitted filename: PLOS_review_SB_Sep_18.docx [file pone.0227875.s003.docx]

“Genetic characterization of Bacillus anthracis strains circulating in Italy from 1972 to 2018” by Rondinone et al. examines B. anthracis strains from the region using well-established PCR based methodologies for bacterial typing of this pathogen. Although there are no novel findings here and despite the fact that the methods applied are being swiftly replaced by newer whole genome sequencing techniques, this study is scientifically sound and does characterize the strains examined based on the most commonly used classification system for this species. This study is largely descriptive in form, with minimal work in terms of computational analyses on the resulting set of genotypes. Nevertheless, I think it would be suitable for publication in PLOS following some serious proofreading and grammatical correction.

The majority of problems with this manuscript are related to the fact that English is a second language for the authors, so many of the suggested changes are grammatical in scope. Nevertheless, I do think there are some over-statements here on the part of the authors which need to be scaled back. In addition, some additional information may be helpful to clarify the findings for the reader.

I did my best to correct the obvious grammatical errors but it may be appropriate for (and in the best interest of) the authors to take this manuscript to a professional English language proofreader/writer to ensure that grammar is correct throughout, as there are many, many mistakes in this manuscript.

Below are my comments/suggestions/edits:

Overall there seem to be a lot places where a sentence ends mid page and the next sentence starts on the following line. It is hard to tell if this is purposeful or some sort of formatting error. Paragraphs should be clearly separated from each other, but otherwise sentences should read from page edge to page edge. This needs to be corrected throughout.

This manuscript is littered with erroneous commas, that are either out of place or just unnecessary.

This manuscript would benefit greatly from some additional computational analysis to slightly expand the methods beyond simple wet-lab typing.

**Abstract:**

Lines 31-33. I find this sentence awkward and suggest rewording it as follows: The phylogenetic identity was determined through the characterization of 14 CanSNPs. In addition, a subsequent 31-loci MLVA assay was also used to further discriminate B. anthracis genotypes into sub-groups.

Lines 33-43. This should read as follows to be grammatically correct: The analysis of 14 CanSNPs allowed for the identification of four main lineages: A.Br.011/009, A.Br. 008/011 (respectively belonging to A.Br. 008/009 sub-lineage, also known Trans-Eurasian or TEA group), A.Br. 005/006 and B. Br. CNEVA.

Line 36. I would change the word “major” to “most common”

Line 37. No need to mention the 31 VNTRs, just say “The MLVA analysis”

Line 38. I do not think you have confirmed a hypothesis. I would say “supporting the hypothesis that all of them are potentially the result of the evolution of a local common ancestral strain”

Line 40-42. I would change this sentence to “The genotyping analysis applied in this study remains a very valuable tool for studying the diversity, evolution, and molecular epidemiology of B. anthracis.” There is no need to keep naming the methods used after establishing them.

**Introduction:**

Line 51. Change “into” to “in”.

Line 52-53. Is there supposed to be a new paragraph here? This seems very awkward to me. Maybe just a formatting error?

Line 53. Change “area” to “areas”.

Line 55: should be “depending on” not “of”.

Line 56: Are both of these types always fatal? I would change to “potentially fatal” if not.

Line 56-57: “Further fatal form” sounds awkward to me, I would find aa way to reword this.

Line 64: there must be something that can be cited to support this, if not how do the authors know?

Line 64: should be “as an epidemic-like disease”

Line 71-73: I would reword this sentence to clearly state your goal for the reader:

The overall goal of this study was to utilize SNP analysis to establish the phylogenetic relationship between the B. anthracis strains examined, and further discriminate them in the context of the MLVA assay, in order to examine correlations among the B. anthracis isolates associated with the Italian anthrax outbreaks and to assess genetic diversity at regional and broader scales.

**Materials and Methods:**

Line 89. I would update this table with a column that includes the regions where the isolates came from

Line 92. Exactly where did this work take place?

Line 98-99. How did you confirm primer amplification? Did you run them on a gel, a genetic analyzer? Give more info here.

Line 119-135. Put the VNTR names and concentrations into a table, this data is too monotonous for the text portion of the manuscript.

I think there needs to be some info added here related to the figure and post wet-lab analysis in general. How was this dataset analyzed? How was the map built?

**Results:**

I think in the Materials and Methods and Results the three PCR based methods should be combined under a single sub-heading (instead of the three: Real Time PCR assay, Canonical Single Nucleotide Polimorphisms (CanSNPs), and Multiple Locus Variable Number Tandem Repeat with 31 VNTRs (MLVA)) and separated simply by paragraph.

Lines 180-186. Is there a way to show the degree of difference between these groups using F_ST_, Nei’s genetic distance or something similar? I think a pairwise matrix that showed differences within and between genotypes would be far more informative then simply listing each genotype with the region and number of isolates.

Again, I think there needs to be some info added here related to the figure and post wet-lab analysis in general as with the methods. How was this dataset analyzed? How was the map built? This ms would benefit greatly from some additional computational analysis.

**Discussion:**

Line 193-194. This sentence is awkward and grammatically incorrect, it should read more like: B. anthracis is clonal in nature, often exhibiting high degrees of genetic homogeneity which has traditionally made it difficult to discriminate for epidemiological purpose.

Line 194-197. Replace multiply with reproduce

Line 197-199. This sentence is grammatically incorrect and it is unclear to me what the authors are trying to say.

Line 200. You say “they appear to be genetically very similar to each other”, but how so? What is the difference in alleles? Be explicit if you can.

Line 211. Although there is definitely evidence to support A.Br. 005/006 is more diffuse in Africa, it is not absent in Europe. It has been documented in Denmark, and the UK.

Line 213. B.Br.CNEVA strains have also been found in the USA, South Africa, and South America, so this group in general is biogeographically diverse, subjugating it to Europe alone is a bit misleading.

Line 219. Caption for Fig. 2. You cannot call this a phylogenetic tree; this tree is based on distances is it not? This is a phylogram then. A phylogeny has a specific definition and therefore the term phylogenetic tree should not be used in this instance.

Also, this figure should be in the results with the methodology for making it the materials and methods section.

Line 237. Need citation here.

Line 247. Should read something like “did not have a greater impact on the genetic structure of B. anthracis in the region”.

Line 248-250 is grammatically incorrect and needs to be rewritten.

Line 255. Grammatic errors here.

Line 259. Should be “the dislocation”

Line 262. Should be “Exclusive to each region”, should be “so it seems”

Line 263. “may be autochthonous” is more appropriate than “are”
