# Supplementary material for: Genetic characterization of Bacillus anthracis strains circulating in Italy from 1972 to 2018
Source: PLoS One. 2020 Jan 13;15(1):e0227875. doi: 10.1371/journal.pone.0227875 (PMC6957342; doi:10.1371/journal.pone.0227875)
Supplement: Attachment — Submitted filename: RESPONSE TO REVIEWERS.docx [file pone.0227875.s006.docx]

**RESPONSE TO REVIEWERS**

**REVIEWER 1**

The document still requires a bit of editorial correction, although vastly improved from the previous round.

The document was further revised from a grammatical point of view.

I still have issue with some of the conclusions made in the discussion portion of the paper. While it is true that the trade history of Italy has been extensive over the centuries, conclusions regarding the genotypes of this study (limited to 1972-2018) cannot be compared to the middle ages unless you have solid data to support it. By the logic of your hypothesis, all isolates can be linked to trade with southern Africa. The time scales with which such genotypic distribution could have occured cannot be determined using MLVA (which inherently includes homoplasy/homoplasticity) and a limited panel of SNPs. Only whole genome sequencing and comparative genomics which includes diverse lineages can provide enough support for such a narrative. There is enough data to recommend this paper without the inclusion of such wild conclusions on the origins of these isolates.

We modified this sentence deleting the part of the middle ages exchanges and making the conclusions more generic.

As to the inclusion of the Simpson's index: In my exprerience, the Shanon diversity better represents allelic diversity in VNTR loci. It will also make your work comparable to other papers.

We replaced the Simpson’s diversity index with the Shannon index as requested.

**REVIEWER 2**

Overall this MS has improved greatly from the previous version, although there is still extensive editing to be done in terms of grammar. I have tried to address all of these issues below but have likely still missed some. I would suggest the authors use the free program/web app Grammarly to specifically address the issue of the overuse of commas throughout. With the incorporation of the edits below I think this manuscript would be suitable for publication in PLOS One.

We further edited the text in terms of grammar and improved the overuse of commas in the text as requested.

Line 51: remove s at the end of “occupations”

Removed

Line 53: add s at the end of “human”.

Added

Line 55: change “a fourth disease form” to “a fourth form of the disease”

Changed in “a fourth form of the disease”

Line 57: change “for use as bacteriological” to “for use as a bacteriological”

Changed in “for use as a bacteriological”

Line 66: remove comma

Removed

Line 75: remove comma after “laboratory”

Removed

Line 130: Remove comma after “analysis” and replace “we used” with “were used”.

Comma removed and “analysis” replaced with “were used”.

Line 139: Table 2: Make sure all rows are equally spaced (rows 6 through 9 are spaced farther apart then the rest)

Rows equally spaced

Lines 157-158: The authors should state what the reference numbers refer to (ncbi or ena, and what specific database)

Reference numbers associated to NCBI database

Line 159: Change “Phylogram”, to “A phylogram”.

Changed

Line 171: Why does sentence end mid-page?

Sentence attached

Line 172: Remove comma

Removed

Line 173: Remove “or”

Removed

Line 174: Remove comma after Europe

Removed

Line 175: Remove both commas surrounding “14th”

Removed

Line 176: change “to” to “the”

Changed

Line 183: remove comma after “VNTRs”

Removed

Line 184: remove comma after “regions”

Removed

Line 198: Table 4: Change “Simpson’s Index of Diversity” to “Simpson's Diversity Index” and throughout.

Sentence reworded as the reviewer 1 asked to use the Shannon diversity index.

Line 202: Remove extreme spacing between words

Removed

Line 207: Change “Around the phylogram are shown, from the external part to the internal part” to “Circling the phylogram from the external to internal region are”

Sentence changed as suggested

Line 214: replace comma with “and”

Replaced

Line 215: change “purpose” to “purposes”

Changed

Line 216-217: I’m not sure this sentence is true, and it is also grammatically incorrect. Does the high survivability of spores lead to genetic homogeneity? I don’t think so, I would argue that genetic homogeneity in this species is due to the fact that it has a single stranded chromosome and reproduces asexually. Persistence of spores in the environment complicate this but is not the defining feature that dictates homogeneity.

Sentence reworded as follows: “*Bacillus anthracis* is clonal in nature and often exhibits a high degree of genetic homogeneity due to the fact that is has a single stranded chromosome and reproduces asexually. This characteristic has traditionally made the discrimination of isolates for epidemiological purposes difficult. Furthermore the high survivability of spores in the soils, allowed *B. anthracis* to reproduce a relatively limited number of times during its evolution”

Line 217-219: This sentence should be reworded to reduce the number of commas and read more coherently.

Sentence reworded as follows: “The 31-loci MLVA analysis carried out on 234 *B. anthracis* strains, isolated in Italy during the years 1972-2018, revealed the circulation of 55 *B. anthracis* genotypes.”

Line 221: Remove comma

Removed

Line 225: Remove comma after “GT-54” and “Veneto”

Removed

Line 226: Remove comma after “observation”

Removed

Line 228: Remove comma after “GT-55”

Removed

Line 229: Remove comma after “Trentino”

Removed

Line 228-229: This sentence is confusing because your allocating a genotype to a genotype. I would reword this to something like: “Furthermore, genotype GT-55; B.Br.CNEVA, isolated in Veneto and Trentino is highly differentiated from most other Italian strains examined here.

Sentence changed as suggested

Line 230: change “branch” to “genotype”, remove “mainly” and change “in particular” to “and found in”

Corrections made as requested

Line 236: Change “this group gave rise” to “this group is thought to have given rise” and cite the paper that originally presented this hypothesis.

Sentence changed as suggested

Line 238: Change “an effect of evolution of a common ancestral strain at territorial level” to “an effect of evolution on a common ancestral strain at the territorial level”

Sentence changed as suggested

Line 240, change “as well as” to “and”

Changed

Line 241: change “seems” to “is postulated”

Changed

Line 247: add the word “strains” after B.Br.CNEVA

“Strains” added after B.Br.CNEVA

Line 248: remove the word “the”

Removed

Line 254: remove comma after “regions” and change “can” to “may”

Comma removed and word changed

Line 256: change “increasing the chances” to “which increases the chances”

Changed

Line 257: Change “this” to “the”

Changed

Line 263: Change “This” to “these”

Changed

Line 264: change space in front of period

Changed
